# Dynamic prognostic nutritional index trajectories predict survival outcomes in nasopharyngeal carcinoma with persistently undetectable plasma Epstein–Barr virus DNA

**DOI:** 10.3389/fnut.2026.1806364

**Published:** 2026-07-03

**Authors:** Ying Li, Qisi Zhang, Honghong Zhang, Zongwei Huang, Jue Wang, Jiajia Zheng, Xiaoke Wang, Xiaoyu Ye, Hanyu Zhang, Youliang Weng

**Affiliations:** 1 Clinical Oncology School of Fujian Medical University, Fujian Cancer Hospital, NHC Key Laboratory of Cancer and Metabolism, Fuzhou, Fujian, China; 2 Department of Radiation Oncology, Xiang'an Hospital of Xiamen University, School of Medicine, Xiamen University, Xiamen, Fujian, China; 3 Fujian Medical University, Fuzhou, Fujian, China

**Keywords:** EBV DNA negative, intensity-modulated radiotherapy, longitudinal trajectory, nasopharyngeal carcinoma, prognostic nutritional index

## Abstract

**Background:**

This study aims to identify prognostic factors in nasopharyngeal carcinoma (NPC) patients with persistently undetectable plasma Epstein–Barr virus (EBV) DNA and to evaluate the association between longitudinal changes in the prognostic nutritional index (PNI) during treatment and survival outcomes.

**Methods:**

We conducted a retrospective cohort study of 412 patients with persistently undetectable plasma EBV DNA NPC. Prognostic factors for progression-free survival (PFS) were identified via Cox regression analyses. A latent class growth mixture model (LCGMM) was performed on serial PNI measurements at baseline (T1), post-induction chemotherapy (T2), and post-radiotherapy (T3) in a subset of 252 patients receiving induction chemotherapy to characterize dynamic nutritional changes. Survival differences between PNI trajectory subgroups were assessed.

**Results:**

Higher baseline PNI was independently associated with improved PFS (HR = 0.93, 95% CI: 0.87–0.98, *p* = 0.008) in patients with persistently undetectable plasma EBV DNA NPC. Longitudinal modeling revealed two distinct PNI trajectory classes in patients receiving induction chemotherapy: a high nutritional reserve group (27%) and a low nutritional reserve (73%) group. The high nutritional reserve type demonstrated superior PFS outcomes in both unadjusted (HR: 0.38, 95% CI: 0.16–0.91, *p* = 0.029) and adjusted models (HR: 0.37, 95% CI: 0.15–0.91, *p* = 0.031). Bootstrap validation supported model stability. No significant association was observed between PNI change rate and PFS (*p* > 0.05).

**Conclusion:**

Our findings demonstrate that baseline PNI is an independent prognostic factor for survival, and that the dynamic PNI trajectory during treatment provides significant prognostic information in NPC patients with persistently undetectable plasma EBV DNA. The identification of distinct nutritional reserve classes through longitudinal modeling provides a novel framework for real-time risk assessment and may help identify patients who could benefit from closer nutritional monitoring and supportive care in future prospective studies.

## 1 Introduction

Nasopharyngeal carcinoma (NPC) is a malignancy that develops in the epithelial cells of the nasopharyngeal mucosa, exhibiting a distinct geographical distribution with particularly high prevalence in East and Southeast Asia (1). According to 2022 GLOBOCAN data, over 120,000 new NPC cases and 70,000 deaths occurred globally, with China accounting for nearly half of these cases (2). Currently, radiotherapy serves as a cornerstone of NPC treatment. In the intensity-modulated radiotherapy (IMRT) era, the 5-year overall survival (OS) rate exceeds 80% (3); however, 10–15% of patients still experience poor prognosis, particularly those with locally advanced disease (4). Clinical prognosis assessment primarily relies on the TNM staging system, yet patients with identical TNM stages often demonstrate heterogeneous outcomes (5). Therefore, precisely predicting NPC prognosis to guide individualized treatment remains a significant clinical challenge.

Tumor biomarkers are essential for early cancer screening and evaluation of treatment efficacy (6). Epstein–Barr virus (EBV) is the most common pathogen associated with NPC (7), and pretreatment plasma EBV DNA levels are clinically utilized as a valuable tool for NPC diagnosis, monitoring, and prognosis assessment (8). However, not all histologically confirmed NPC patients exhibit detectable EBV DNA (9), with a subset presenting persistently undetectable EBV DNA throughout the entire treatment process. Our previous work identified that this subgroup (U–U–U) exhibits a favorable prognosis, with a 5-year overall survival rate of 86.3% (10). Nevertheless, the absence of this dynamic circulating marker creates a distinct challenge for the ongoing risk stratification and management of these patients. Thus, exploring surrogate predictive biomarkers for persistently plasma EBV DNA-undetectable NPC patients holds significant clinical implications for optimizing the prognostic evaluation framework in this distinct subpopulation.

Immunonutritional status correlates with tumor prognosis. The prognostic nutritional index (PNI), calculated by integrating serum albumin and peripheral lymphocyte count, serves as an effective indicator reflecting patients’ nutritional and inflammatory status (11). It has been established as an independent prognostic factor for multiple solid tumors (12). Previous studies have investigated the impact of pre-treatment PNI on NPC prognosis (13–15), consistently demonstrating its promise as a prognostic biomarker. Although prior research has explored the prognostic value of certain immunonutritional indicators in NPC patients with persistently undetectable plasma EBV DNA (16), the prognostic significance of PNI—a core biomarker comprehensively reflecting nutritional reserves and immune function—and its dynamic changes during treatment remain unelucidated in this distinct subpopulation.

In this study, we aim to clarify the prognostic value of the PNI level and its longitudinal dynamic changes in NPC patients with persistently undetectable plasma EBV DNA, to provide a novel prognostic assessment tool for this subset lacking effective biomarkers.

## 2 Materials and methods

### 2.1 Patients and data collection

This retrospective observational study was approved by the Institutional Research Ethics Board of Fujian Cancer Hospital (approval number: K2024-289-01). Consecutive patients were identified, where baseline characteristics and outcomes were recorded. The inclusion criteria were as follows: (1) histopathologically confirmed NPC (keratinizing or non-keratinizing squamous cell carcinoma); (2) persistently undetectable EBV DNA during treatment; (3) complete medical records and peripheral blood test data; (4) received radical IMRT; and (5) had no evidence of disease progression (locoregional recurrence or distant metastasis) or death prior to radiotherapy completion. Exclusion criteria included: (1) treatment interruption; (2) undocumented clinical stage; and (3) loss to follow-up, defined as a post-radiotherapy observation period of less than 3 months. All patients were staged via clinical examination and computed tomography (CT) or magnetic resonance imaging (MRI) of the head and neck and thorax, with or without fluorodeoxyglucose positron emission tomography/computed tomography (^18^F-FDG PET/CT). Patients were restaged for analysis using the American Joint Committee on Cancer (AJCC) 8th edition by two experienced radiation oncologists. The requirement for informed consent was waived due to the retrospective and de-identified nature of the data. This study adhered to the Strengthening the Reporting of Observational Studies in Epidemiology (STROBE) Statement.

The immune-inflammatory and nutritional indicators analyzed included PNI, neutrophil-lymphocyte ratio (NLR), platelet-lymphocyte ratio (PLR), monocyte-lymphocyte ratio (MLR), systemic immune-inflammation index (SII), systemic inflammation response index (SIRI), and pan-immune-inflammation value (PIV). PNI was calculated as serum albumin concentration (g/L) + 5 × total lymphocyte count (10^9^/L). The collection of hematological parameters and the formulas for other indicators were detailed in the Supplementary Methods.

### 2.2 Plasma EBV DNA quantitation

Plasma EBV DNA levels were measured longitudinally from admission to post-radiotherapy. A minimum of three measurements were obtained per patient: at admission (T0), after induction chemotherapy (IC) or immediately before radiotherapy initiation (T1), and upon radiotherapy completion (T2). Patients were classified as having plasma EBV DNA-undetectable NPC if viral loads remained at 0 copies/mL at all three time points. EBV DNA was quantified using a real-time quantitative polymerase chain reaction assay, with detailed methodological descriptions provided previously (10).

### 2.3 Treatment

The IMRT was administered to all patients. Patients with Stage I received IMRT alone; patients with Stage II-IVa received radiotherapy plus chemotherapy. Radical IMRT spanned 6–7 weeks, delivering a median radiation dose of 69.96 Gy (IQR, 69.70–70.00) to the primary tumor and 69.75 Gy (IQR, 68.25–70.00) to involved neck lymph nodes. IC regimens mainly consisted of platinum combined with gemcitabine or paclitaxel, administered intravenously every 3 weeks for 1–6 cycles. Concurrent chemotherapy involved single-agent platinum administered intravenously every 3 weeks for 1–3 cycles. The use of adjuvant chemotherapy was determined by the treating clinician based on the patient’s condition. Deviations from the guidelines were due to participation in clinical trials, patient refusal, advanced age, or organ dysfunction, suggesting potential intolerance to treatment. Salvage treatments were provided as appropriate for patients with relapse or metastatic disease.

### 2.4 Follow-up and endpoints

Patients were followed up every 3 months for the first 2 years, every 6 months from years 3 to 5, and subsequently on an annual basis. The follow-up evaluations included: physical examination, nasopharyngoscopy, plasma EBV DNA quantification, routine hematological tests, abdominal ultrasonography, chest CT, head and neck MRI (once every 6 months), and whole-body bone scan (once a year). The final follow-up date was March 15, 2025. The primary endpoint was progression-free survival (PFS), defined as the time from the end of radiotherapy to the earliest occurrence of disease progression, including all-cause death, locoregional recurrence, or distant metastasis.

### 2.5 Latent class growth mixture model (LCGMM) analysis

To identify distinct longitudinal patterns of the PNI during treatment, we performed an LCGMM using the lcmm R package. To mitigate bias in trajectory fitting, we restricted the analysis to patients who received IC and had complete PNI data at three time points: baseline (T1), post-IC (T2) and post-RT (T3). PNI was modeled as a function of time within a linear mixed-effects framework, allowing patients to be classified into heterogeneous subgroups through multinomial logistic regression. We specified a parsimonious random-effects structure—random intercepts only—to ensure model identifiability and avoid overfitting given the limited number of time points. Class-specific linear time effects were estimated through the mixture component, permitting distinct trajectory classes to differ in both intercept and rate of change. Model selection considered linear and quadratic specifications for one to three latent classes. Deterministic starting values were used, with the k-class model initialized using estimates from the (k-1)-class model, the default and recommended approach in lcmm. The optimal number of latent classes was determined using the following criteria (17): (1) lowest Bayesian information criterion (BIC); (2) average posterior probability > 70% for each class; and (3) each class comprising at least 5% of the sample. Classification quality was assessed by average posterior probabilities (>70% indicates good classification) and by the odds of correct classification (OCC = [*P*_k_/(1 − *P*_k_)]/[π_k_/(1 − π_k_)], where *P*_k_ is the average posterior probability and π_k_ is the class proportion); OCC > 5 denotes excellent classification. Model convergence was evaluated using parameter change (<1 × 10^−4^) and log-likelihood change (<1 × 10^−4^). Internal validation was performed by bootstrap resampling with 200 iterations, from which bootstrap-adjusted hazard ratios (HRs) and 95% confidence intervals (CIs) were derived.

### 2.6 Statistical analysis

Baseline characteristics were analyzed using descriptive statistics, with categorical variables presented as frequencies and percentages, while continuous variables were presented as medians and interquartile ranges (IQRs). Categorical variables were compared using Chi-square tests, and continuous variables were compared via Mann–Whitney *U* tests. PFS rates were calculated using the Kaplan–Meier method, and differences in survival curves between different subgroups were compared using the log-rank test. Median follow-up time was estimated using the reverse Kaplan–Meier method. Univariate Cox proportional hazards regression identified potential prognostic factors. The proportional hazards (PH) assumption was tested for all variables using Schoenfeld residuals. Collinearity among variables was assessed using the variance inflation factor (VIF), with a threshold of >5 indicating significant collinearity. Variables with *p* < 0.05 were included in the multivariate Cox model to determine independent predictors. Variables violating the PH assumption were noted but did not influence model selection if they already failed to reach significance in univariate analysis. To assess the incremental prognostic value of PNI trajectories, we compared our model against established NPC prognostic markers, including TNM stage, SIRI and PIV (18–20). A clinical reference model was constructed using T and N categories (the standard clinical staging system). Model performance was compared using Akaike information criterion (AIC), Harrell’s C-index and net reclassification improvement (NRI). Likelihood ratio tests were used to determine whether the addition of PNI trajectory significantly improved model fit. Detailed procedures for the NRI analysis are provided in the Supplementary Methods. Multivariable Cox proportional hazards regression was used to assess the association between PNI trajectory class and PFS, adjusting for baseline covariates that differed significantly between the two trajectory groups (age and sex). Baseline PNI was not included as a covariate because it formed part of the trajectory definition. Other clinical covariates, such as T category, N category, histological subtype and concurrent chemotherapy, were not included, as they did not differ significantly between trajectory groups and therefore did not satisfy the criteria for confounders in this analysis. Cox regression was also used to evaluate the impact of the rate of PNI change on PFS and to assess the association between trajectory class and PFS across different subgroups. ΔPNI was calculated as the change in PNI between consecutive time points: for patients who received IC, from baseline to post-IC (ΔPNI_1_) and from post-IC to radiotherapy completion (ΔPNI_2_); for those who did not receive IC, from baseline to radiotherapy completion.

All analyses were conducted in R (v4.0.4; R Project for Statistical Computing, RRID: SCR_001905), along with Zstats (v1.0; www.zstats.net) and SPSS Statistics (v25.0; IBM SPSS Statistics, RRID: SCR_016479), with statistical significance defined as two-sided *p* < 0.05.

## 3 Results

### 3.1 Patient characteristics

We reviewed 462 newly diagnosed non-metastatic NPC patients with negative EBV DNA between January 2016 and December 2022 at our institution. All patients had keratinizing or non-keratinizing squamous cell carcinoma. Following predefined exclusion criteria, 50 patients were excluded, including 24 with treatment abandonment, 18 with unknown T or N category, and 8 lost to follow-up. The study cohort finally comprised 412 patients, of whom 213 (51.7%) were younger than 50 years and 199 (48.3%) were 50 years or older. The majority of patients were male (71.8%). Histologically, most patients (98.3%) had non-keratinizing carcinoma, while only 7 (1.7%) had keratinizing squamous cell carcinoma. EBV-encoded small RNA (EBER) data were available for 227 patients (55.1%). Of these, 203 (89.4%) were EBER-positive and 24 (10.6%) were EBER-negative. More than half of the patients were seen in the advanced stage at diagnosis (61.9%). Approximately 61.2% (*n* = 252) of patients underwent IC, and 61.4% (*n* = 253) received concurrent chemotherapy. During a median follow-up of 52.9 months (95%CI: 49.5–55.4 months), a total of 50 progression events were documented, including 28 locoregional recurrences, 16 distant metastases, and 29 deaths (some patients experienced multiple event types). The 5-year PFS rate of these patients was 86.7% (95% CI: 82.9–90.7%). The median values of hematological indicators at admission were as follows: NLR, 1.92 (IQR 1.50–2.67), PLR, 122.02 (IQR 96.29–157.65), MLR, 0.19 (IQR 0.15–0.26), SII, 461.07 (IQR 338.49–632.92), SIRI, 0.75 (IQR 0.51–1.10), PIV, 176.94 (IQR 104.40–273.17), and PNI, 52.50 (IQR 49.10–56.10). Other baseline characteristics of the patients are presented in Table 1.

**Table 1:** Baseline characteristics of NPC patients with negative EBV DNA (*N* = 412).

Characteristic	No. (%)/median (IQR)
Age, years	
<50	213 (51.7)
≥50	199 (48.3)
Sex	
Male	296 (71.8)
Female	116 (28.2)
Histological subtype	
Nonkeratinizing carcinoma	405 (98.3)
Keratinizing carcinoma	7 (1.7)
EBER status	
Positive	203 (49.3)
Negative	24 (5.8)
Missing	185 (44.9)
T category	
T1-2	217 (52.7)
T3-4	195 (47.3)
N category	
N0-1	298 (72.3)
N2-3	114 (27.7)
TNM-8 stage	
Stage I–II	157 (38.1)
Stage III–IVa	255 (61.9)
GTV-T, Gy (IQR)	69.96 (69.70, 70.00)
GTV-N, Gy (IQR)	69.75 (68.25, 70.00)
Induction chemotherapy	
No	160 (38.8)
Yes	252 (61.2)
Concurrent chemotherapy	
No	159 (38.6)
Yes	253 (61.4)
NLR (IQR)	1.92 (1.50, 2.67)
PLR (IQR)	122.02 (96.29, 157.65)
MLR (IQR)	0.19 (0.15, 0.26)
SII (IQR)	461.07 (338.49, 632.92)
SIRI (IQR)	0.75 (0.51, 1.10)
PIV (IQR)	176.94 (104.40, 273.17)
PNI (IQR)	52.50 (49.10, 56.10)
5-year PFS, actuarial rate (95% CI), %	86.7 (82.9, 90.7)

Continuous variables were represented as median with IQR; while categorical data were represented as absolute values and proportions.

EBER, Epstein–Barr virus-encoded small RNA; EBV DNA, Epstein–Barr virus DNA; GTV-N, irradiation dose of gross tumor volume of lymph nodes; GTV-T, irradiation dose of gross tumor volume of primary nasopharyngeal tumor; IQR, interquartile range; MLR, monocyte-lymphocyte ratio; NLR, neutrophil-lymphocyte ratio; NPC, nasopharyngeal carcinoma; PFS, progression free survival; PIV, pan-immune-inflammation value; PLR, platelet-lymphocyte ratio; PNI, prognostic nutritional index; SII, systemic immune-inflammation index; SIRI, systemic inflammation response index.

### 3.2 Identification of prognostic factors in persistently undetectable EBV DNA NPC patients

We evaluated several parameters to identify potential prognostic factors of PFS in NPC patients with persistently undetectable plasma EBV DNA (Table 2). The results of the PH assumption test are shown in Supplementary Table 1. Among these variables, univariate analysis revealed that age (HR: 2.45, 95% CI: 1.33–4.49, *p* = 0.004), histological subtype (HR: 11.29, 95% CI: 3.97–32.09, *p* < 0.001), N category (HR: 3.19, 95% CI: 1.82–5.60, *p* < 0.001), IC (HR: 4.32, 95% CI: 1.94–9.61, *p* < 0.001), and baseline PNI (HR: 0.89, 95% CI: 0.84–0.94, *p* < 0.001) significantly affected PFS in univariate analysis. Multivariate analysis further indicated that age ≥ 50 years (HR: 2.11, 95% CI: 1.13–3.95, *p* = 0.019), keratinizing carcinoma (HR: 9.20, 95% CI: 3.11–27.20, *p* < 0.001), N2-3 (HR: 2.14, 95% CI: 1.17–3.95, *p* = 0.014), receipt of IC (HR: 2.66, 95% CI: 1.12–6.30, *p* = 0.027) were identified as independent risk factors for poor PFS, while a higher baseline PNI (HR: 0.93, 95% CI: 0.87–0.98, *p* = 0.008) was an independent protective factor for PFS outcomes in these patients. Patients who received IC had significantly more advanced disease, including higher T category (T3–4: 73.4% vs. 6.3%, *p* < 0.001), higher N category (N2–3: 43.7% vs. 2.5%, *p* < 0.001), and lower baseline PNI (*p* = 0.015), consistent with confounding by indication (Supplementary Table 2). Analysis of patients receiving IC (*n* = 252) confirmed the robustness of PNI as an independent prognostic factor for PFS (HR: 0.93, 95% CI: 0.88–0.99, *p* = 0.038, Supplementary Tables 3, 4). Collinearity diagnostics showed no significant multicollinearity among the included variables (Supplementary Tables 5, 6).

**Table 2:** Identification of prognostic factors of PFS in NPC patients with negative EBV DNA by univariate and multivariate Cox regression analysis.

Characteristic	Univariate		Multivariate	
	HR (95% CI)	*p*-value	HR (95% CI)	*P*-value
Age, years				
<50	1.00 (Reference)		1.00 (Reference)	
≥50	2.45 (1.33–4.49)	0.004	2.11 (1.13–3.95)	0.019
Sex				
Male	1.00 (Reference)			
Female	1.19 (0.65–2.16)	0.575		
Histological subtype				
Nonkeratinizing carcinoma	1.00 (Reference)		1.00 (Reference)	
Keratinizing carcinoma	11.29 (3.97–32.09)	<0.001	9.20 (3.11–27.20)	<0.001
T category				
T1-2	1.00 (Reference)			
T3-4	1.74 (0.98–3.08)	0.057		
N category				
N0-1	1.00 (Reference)		1.00 (Reference)	
N2-3	3.19 (1.82–5.60)	<0.001	2.14 (1.17–3.95)	0.014
GTV-T, Gy	0.79 (0.05–12.66)	0.865		
GTV-N, Gy	1.00 (1.00–1.00)	0.157		
Induction chemotherapy				
No	1.00 (Reference)		1.00 (Reference)	
Yes	4.32 (1.94–9.61)	<0.001	2.66 (1.12–6.30)	0.027
Concurrent chemotherapy				
No	1.00 (Reference)			
Yes	1.39 (0.75–2.59)	0.298		
NLR	1.14 (0.96–1.37)	0.144		
PLR	1.00 (1.00–1.01)	0.148		
MLR	1.27 (0.63–2.57)	0.503		
SII	1.00 (1.00–1.00)	0.086		
SIRI	1.03 (0.87–1.22)	0.724		
PIV	1.00 (1.00–1.00)	0.140		
PNI	0.89 (0.84–0.94)	<0.001	0.93 (0.87–0.98)	0.008

EBV, Epstein–Barr virus; GTV-N, irradiation dose of gross tumor volume of lymph nodes; GTV-T, irradiation dose of gross tumor volume of primary nasopharyngeal tumor; MLR, monocyte-lymphocyte ratio; NLR, neutrophil-lymphocyte ratio; NPC, nasopharyngeal carcinoma; PFS, progression free survival; PIV, pan-immune-inflammation value; PLR, platelet-lymphocyte ratio; PNI, prognostic nutritional index; SII, systemic immune-inflammation index; SIRI, systemic inflammation response index.

### 3.3 PNI trajectory analysis

**Figure 1:**
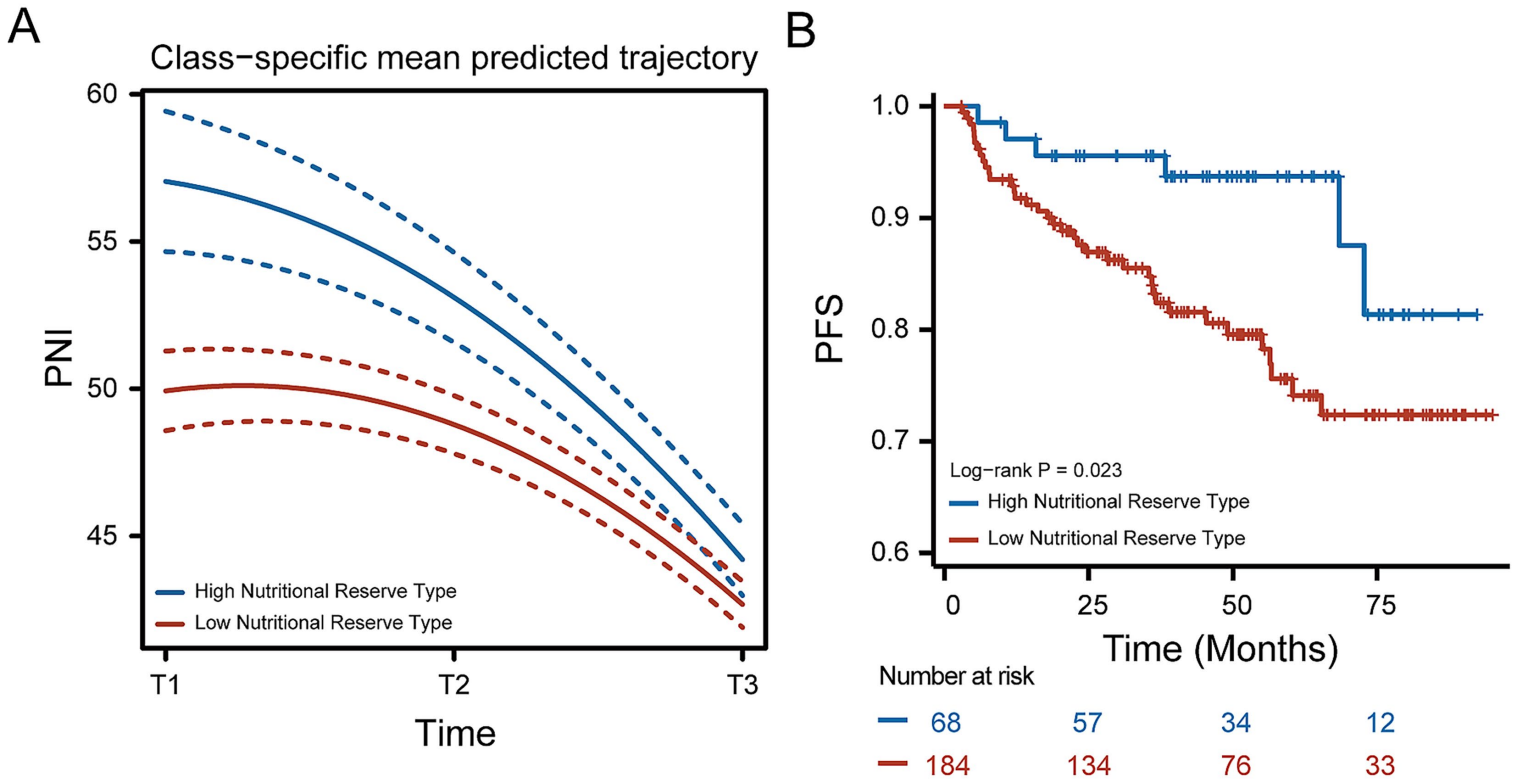
Trajectories of PNI and survival outcomes in NPC patients with induction-concurrent (chemo)radiotherapy. **(A)** Predicted trajectories of prognostic nutritional index (PNI) identified by group-based trajectory modeling. **(B)** Kaplan–Meier curves of progression-free survival (PFS) according to PNI trajectory group. NPC, nasopharyngeal carcinoma; PFS, progression-free survival; PNI, prognostic nutritional index.

Given the prognostic effect of baseline PNI in patients with persistently undetectable plasma EBV DNA, we further explored whether longitudinal changes in PNI during treatment might affect patient outcomes. Longitudinal PNI measurements were obtained at three predefined intervals. To identify the optimal trajectory structure, we constructed an LCGMM using linear and quadratic polynomial functions. The quadratic two-class solution was selected as optimal based on the fit indices shown in Table 3. This model converged successfully after 11 iterations (parameter change: 1.7 × 10^−7^; log-likelihood change: 3.2 × 10^−8^), indicating stable estimation. The trajectories of PNI during treatment are illustrated in Figure 1A and Supplementary Figure 1, and were labeled as “high nutritional reserve” type (*n* = 68, 27.0%) and “low nutritional reserve” type (*n* = 184, 73.0%) according to their shapes. PNI levels in both groups declined as treatment progressed. The analysis of baseline characteristics between PNI trajectory subgroups (Supplementary Table 7) identified statistically significant intergroup disparities exclusively in age and sex distribution (*p* < 0.05 for both). In the high-reserve group, six progression events were observed (four locoregional recurrences, two distant metastases and three deaths), whereas the low-reserve group experienced 37 events (22 locoregional recurrences, 13 distant metastases and 22 deaths), with some patients experiencing multiple event types.

**Table 3:** Fit indices for latent class growth models of PNI.

No. Latent class	Polynomial degree	Log-Lik	BIC	Proportion of participants per class (%)	Mean posterior probabilities (%)	Posterior probabilities > 70% (%)	OCC
1	Linear	−2193.09	4407.00	100.0	NA	NA	NA
1	Quadratic	−2152.15	4331.95	100.0	NA	NA	NA
2	Linear	−2181.38	4407.00	71.8/28.2	86.9/75.0	82.3/60.6	2.6/7.6
**2**	**Quadratic**	**−2135.29**	**4320.34**	**73.0/27.0**	**87.4/76.9**	**85.9/64.7**	**2.6/9.0**
3	Linear	−2177.57	4421.49	29.8/57.1/13.1	80.5/71.5/77.9	72.0/66.0/75.8	9.72/1.9/23.4
3	Quadratic	−2131.41	4334.69	24.2/63.5/12.3	80.0/73.7/79.4	72.1/69.4/71.0	12.5/1.6/27.5

BIC, Bayesian Information Criterion; Log-Lik, the maximum Log-Likelihood; NA, not applicable; OCC, odds of correct classification; PNI, prognostic nutritional index.

The best fitting model is highlighted in bold characters.

### 3.4 PNI trajectories inform on prognosis in persistently undetectable EBV DNA NPC patients

To characterize the relationship between the dynamics of PNI during treatment and PFS, Kaplan–Meier analysis showed that the high nutritional reserve type had superior 5-year PFS (93.6, 95% CI: 87.8–100.0%) compared to the low nutritional reserve type (75.3, 95% CI: 68.1–83.4%) (*p* = 0.023) (Figure 1B). After adjusting for age and sex, multivariate Cox regression analysis identified significant associations between PNI trajectories and PFS outcomes (Table 4). Specifically, the high nutritional reserve type demonstrated superior PFS outcomes compared to the low-reserve one in both unadjusted (HR: 0.38, 95% CI: 0.16–0.91, *p* = 0.029) and adjusted models (HR: 0.37, 95% CI: 0.15–0.91, *p* = 0.031). Bootstrap internal validation with 200 resamples demonstrated excellent model stability; all replicates converged and 100% supported the two-trajectory solution. The bootstrap-adjusted HR (high vs. low reserve) remained 0.37 (95% bootstrap CI: 0.15–0.91), consistent with the original estimate (Supplementary Table 8).

**Table 4:** Association of PNI trajectory and PFS in NPC patients by Cox proportional hazard regression analysis.

Characteristic	Model 1		Model 2	
	HR (95% CI)	*P*-value	HR (95% CI)	*P*-value
PNI trajectory				
Low Nutritional Reserve Type	1.00 (Reference)	0.029	1.00 (Reference)	0.031
High Nutritional Reserve Type	0.38 (0.16–0.91)		0.37 (0.15–0.91)	

Model 1 was with no covariate.

Model 2 was adjusted for age, and sex.

NPC, nasopharyngeal carcinoma; PFS, progression-free survival; PNI, prognostic nutritional index.

To assess the independent incremental prognostic value of PNI trajectory beyond established clinical and inflammatory markers, we compared four nested Cox regression models (Supplementary Table 9). Model 2 (clinical model plus PNI trajectory) outperformed the clinical model alone (Model 1), with a lower AIC (427.92 vs. 431.10), a higher C-index (0.63, 95% CI: 0.59–0.68 vs. 0.56, 95% CI: 0.52–0.61) and a positive net reclassification improvement (NRI = 0.16, 95% CI: 0.01–0.30). A likelihood ratio test confirmed that the addition of PNI trajectory significantly improved the model fit (*p* = 0.021). Adding PNI trajectory to the combined clinical-inflammation model (Model 4 vs. Model 3) also improved model fit, although the change did not reach statistical significance (AIC: 431.03 vs. 433.95; C-index: 0.66, 95% CI: 0.61–0.70 vs. 0.59, 95% CI: 0.54–0.64; *p* = 0.060).

### 3.5 Subgroup analyses of the association between PNI trajectory and PFS

To further assess the robustness of the association between PNI trajectory and PFS, we performed subgroup analyses. As shown in Supplementary Figure 2, the protective association of the high nutritional reserve trajectory reached statistical significance in male patients (HR = 0.30, 95% CI: 0.10–0.86, *p* = 0.025) and patients with stage III–IVa disease (HR = 0.33, 95% CI: 0.13–0.85, *p* = 0.021), and showed marginal significance in those with non-keratinizing subtype (HR = 0.43, 95% CI: 0.18–1.02, *p* = 0.056), T3–4 category (HR = 0.36, 95% CI: 0.12–1.02, *p* = 0.054), and N2–3 category (HR = 0.35, 95% CI: 0.10–1.16, *p* = 0.087). The consistently directionally protective HRs across all subgroups (all HRs < 1.0) and the non-significant interaction terms support the robustness of the association between high nutritional reserve trajectory and improved PFS across the IC-treated population.

The rate of PNI change showed no significant association with PFS, whether assessed as ΔPNI_1_ (baseline to post-IC) or ΔPNI_2_ (post-IC to radiotherapy completion), in either the overall cohort (n = 252) or within trajectory subgroups (Table 5). Similarly, in the 160 patients who did not receive IC, ΔPNI from baseline to radiotherapy completion was not associated with PFS (HR = 1.01, 95% CI: 0.87–1.17, *p* = 0.872).

**Table 5:** Univariate Cox regression analysis of PNI change rate and PFS in NPC patients with induction-concurrent (chemo)radiotherapy.

Variable	HR (95%CI)	*p*-value	*P* for interaction
ΔPNI_1_			
All patients	4.94 (0.21–116.13)	0.321	
PNI trajectory			0.409
Low Nutritional Reserve Type	1.18 (0.04–37.62)	0.927	
High Nutritional Reserve Type	65.40 (0.01–695081.34)	0.377	
ΔPNI_2_			
All patients	5.98 (0.20–179.76)	0.303	
PNI trajectory			0.125
Low Nutritional Reserve Type	5.92 (0.14–258.57)	0.356	
High Nutritional Reserve Type	0.01 (0.00–211.50)	0.343	

ΔPNI_1_, PNI change rate between T1 and T2; ΔPNI_2_, PNI change rate between T2 and T3; NPC, nasopharyngeal carcinoma; PFS, progression free survival; PNI, prognostic nutritional index.

## 4 Discussion

To our knowledge, this study represents the first application of LCGMM to delineate the dynamic trajectories of PNI in patients with persistently plasma EBV DNA-undetectable NPC and to evaluate their prognostic utility. In a retrospective cohort of 412 patients, baseline PNI was validated as an independent prognostic factor for PFS. We identified two distinct phenotypic trajectories in the subgroup of patients who received IC: high and low nutritional reserve pattern. In particular, the trajectory characterized by low nutritional reserve was significantly associated with unfavorable outcomes. Furthermore, we observed that the rate of PNI change was not correlated with PFS, reaffirming the significance of baseline nutritional reserve for the prognosis in this patient population.

The prognostic assessment and formulation of treatment strategies for NPC are largely dependent on effective biomarkers. The development of NPC is closely associated with EBV infection (7). Consequently, plasma EBV DNA load is a well-established key biomarker for NPC, crucial for prognostic evaluation and therapeutic decision-making (8). However, a specific patient subgroup exists in clinical practice that maintains a persistently negative plasma EBV DNA status throughout the entire course of diagnosis and treatment. The prognostic characteristics of this subgroup remain controversial, with inconsistent findings in the existing literature. Several studies have indicated that patients with persistently undetectable EBV DNA status have significantly superior survival outcomes compared to their positive counterparts, which may be attributed to the higher propensity for distant metastasis in EBV DNA-positive patients (21–24). In contrast, a prospective study by Nicholls et al. in an endemic population demonstrated that the survival outcomes of negative plasma EBV DNA patients were not superior to those of positive EBV DNA patients (25). Furthermore, a study by Huang et al. also confirmed that the PFS of patients who were persistently undetectable EBV DNA (the U–U–U group) showed no significant advantage over that of patients who became negative after IC (the D-U–U group) (10). These disparate findings underscore the challenge of performing precise risk stratification for this population. Therefore, identifying alternative prognostic biomarkers applicable to persistently plasma EBV DNA-undetectable patients holds significant clinical implications.

In this study, we focused on NPC patients with persistently negative plasma EBV DNA. We acknowledge that EBER *in situ* hybridization is the gold standard for confirming EBV association at the tissue level (26). However, we chose to define our cohort based on plasma EBV DNA status for two reasons. First, plasma EBV DNA is widely used in clinical practice for diagnosis, monitoring, and risk stratification, making our findings directly relevant to clinical decision-making. Second, EBER-negative NPC is extremely rare (1.7% in our institutional cohort of 2,942 patients) (27); restricting the analysis to EBER-negative patients would yield an insufficient sample size for robust statistical analysis. Notably, nearly half of our cohort (49.3%) were EBER-positive despite being persistently undetectable EBV DNA, underscoring the complexity of EBV status determination and representing an interesting subgroup for future research.

To address the prognostic challenge in this heterogeneous population, previous studies have begun to explore various baseline, host-related alternative prognostic indicators. For instance, Yuan et al. systematically evaluated several inflammation-based markers and identified SIRI as a robust independent prognostic factor for this subgroup (28). More recently, Weng et al. (16) integrated multiple parameters using principal component analysis to construct a comprehensive immune-inflammation index and nutrition index, further validating the value of assessing host status at baseline. These investigations provide valuable insights into the field and consistently point toward the importance of evaluating the host’s immune-inflammatory status.

The prognosis of cancer patients is influenced by their immune and nutritional status (29, 30). Consequently, the PNI, which integrates these two factors, has emerged as a research hotspot in cancer prognosis in recent years. PNI was initially proposed by Onodera et al. for assessing the postoperative nutritional status of patients with gastric cancer (31). In recent years, its prognostic value has been validated across numerous tumor types (32–35). In the context of NPC, the value of PNI as a potential biomarker was first elucidated by the work of Du et al. (36). They discovered that pre-treatment PNI holds significant predictive value for OS, distant metastasis-free survival, and PFS in NPC patients. Subsequently, a retrospective study by Jiang et al. (37) involving 618 patients corroborated these findings and established an optimal PNI cutoff value of 48.1. However, for the specific subgroup of patients with persistently undetectable EBV DNA status, the dynamic evolution of the PNI throughout the entire therapeutic course and its potential value as a longitudinal biomarker has yet to be elucidated.

In the present study, we confirmed that baseline PNI is an independent prognostic factor for PFS in persistently plasma EBV DNA-undetectable patients, which is consistent with the findings of Du et al. (36) and Jiang et al. (37) in the general NPC population. The underlying mechanism may be attributable to the complex interplay between the tumor and the host. PNI is a parameter based on both nutritional and inflammatory status, calculated from the serum albumin level and the total lymphocyte count (38). It is well established that serum albumin is not only an indicator of nutritional status but is also closely associated with the systemic inflammatory response (12). In chronic inflammatory diseases such as cancer, elevated levels of pro-inflammatory cytokines can suppress hepatic albumin synthesis (39). Therefore, hypoalbuminemia not only reflects the body’s nutritional depletion but also serves as a crucial measure of the host’s inflammatory response intensity, and it is significantly associated with disease progression and adverse prognosis (40). On the other hand, lymphocytes are an essential component of the nonspecific immune system and play a vital role in host anti-tumor immunity. They can inhibit the growth and dissemination of cancer cells by mediating cytotoxic effects and producing cytokines (41). Tumor cells, however, can reshape their microenvironment through various mechanisms, thereby constructing a complex network that systematically suppresses the immune response (42). Consequently, a low peripheral lymphocyte level may indicate a compromised lymphocyte-mediated anti-tumor immune response and suggest a poorer cancer prognosis (43). Taken together, baseline PNI can be regarded as an objective indicator that comprehensively reflects a patient’s nutritional reserve and immune capacity before treatment, two factors that collectively influence the survival outcomes of cancer patients.

The single-timepoint biomarker assessments cannot capture the dynamic physiological processes that dictate disease progression and treatment response. While Küçükarda et al. (44) advanced the field by establishing both pre- and post-treatment PNI as independent prognostic factors for OS in non-metastatic NPC, their analysis remained constrained to a single-timepoint assessment and did not take into account the dynamic changes of PNI during treatment. Here, we modeled the longitudinal trajectory of PNI throughout treatment in patients receiving IC using LCGMM and identified two distinct profiles—a “high nutritional reserve” trajectory and a “low nutritional reserve” trajectory—with profoundly divergent prognostic outcomes. Multivariate analysis showed that the PNI trajectory classification was significantly associated with PFS. Bootstrap internal validation also supported the stability of the two-trajectory solution (100% bootstrap replicates). The bootstrap-adjusted HR remained significant, although the mean proportion of the high-reserve group estimated by bootstrap (39.9%) was higher than the original estimate (27.0%), with a wide 95% CI (17.8–61.9%). This uncertainty in class proportion estimation probably reflects the limited sample size of the high-reserve group (*n* = 68), while also highlighting the need for external validation in larger cohorts. Our model comparison analysis demonstrated that PNI trajectory classification provides independent incremental prognostic value beyond established clinical (T and N categories) and inflammatory (SIRI, PIV) markers. The addition of PNI trajectory significantly improved model fit, as reflected by lower AIC and higher C-index. These findings suggest that longitudinal assessment of nutritional reserve captures aspects of host status that are not fully accounted for by single-timepoint clinical or inflammatory markers. Notably, some conventional prognostic factors, such as TNM stage, did not reach statistical significance in our persistently undetectable EBV DNA cohort. This observation is consistent with previous reports (28) suggesting that the prognostic impact of anatomical staging may be attenuated in persistently plasma EBV DNA-undetectable NPC, further highlighting the need for alternative biomarkers tailored to this specific patient subgroup and supporting the clinical utility of PNI trajectory as a novel prognostic tool in this population.

Our findings suggest that cumulative nutritional and immunologic reserve is one of the critical determinants of prognosis in NPC patients with persistently undetectable plasma EBV DNA. The rate of PNI change showed no significant association with PFS across all subgroups examined, suggesting that the absolute level of nutritional reserve—particularly its persistence throughout treatment—has greater prognostic value than transient fluctuations. This finding also addresses a potential concern that the identified trajectories might merely reflect the short-term nutritional impact of induction chemotherapy, as such an effect would likely manifest as a correlation between ΔPNI and outcomes. Several factors may explain the absence of a significant association between ΔPNI and PFS. First, the timing of PNI measurements may not capture acute inflammatory phases or rapid nutritional fluctuations that could occur between assessments. Second, PNI is a composite indicator of nutritional and immunologic status that changes relatively slowly during treatment, reflecting cumulative host reserve rather than acute-phase responses. Consequently, transient fluctuations in PNI may have limited prognostic value compared with the sustained trajectory pattern. The observed prognostic impact of PNI trajectories probably arises from their ability to identify patients with persistently low nutritional reserve from baseline to treatment completion. These patients have significantly worse PFS, with no evidence of “catch-up” during treatment. Therefore, the prognostic impact of PNI trajectories is likely mediated by a sustained depression in PNI, reflecting protracted immunodeficiency and catabolic states that collectively foster a tumor-permissive milieu (45). These results suggest that, in clinical practice, the focus of prognostic assessment should shift from short-term fluctuations of PNI to its overall reserve level maintained throughout the entire course of treatment. PNI trajectories may help identify patients at risk of diminished nutritional reserves who warrant closer nutritional assessment. However, whether early nutritional intervention would improve outcomes in these patients remains to be established in future prospective interventional studies. Future studies with more frequent sampling intervals and concurrent measurement of acute inflammatory markers (e.g., IL-6, CRP) are warranted to further explore these dynamics.

Several methodological considerations regarding the LCGMM warrant discussion. First, classification quality was suboptimal for certain metrics: the OCC for the low-reserve class was 2.6 (below the recommended threshold of 5), and only 64.7% of patients in the high-reserve class had a posterior probability exceeding 70%. This uncertainty probably reflects the limited number of time points (three PNI measurements) and the modest size of the high-reserve subgroup (*n* = 68), rather than inherently poor class separation. Second, we adopted a parsimonious random-effects structure (random intercepts only) to avoid overfitting. More complex structures, such as the inclusion of random slopes, led to non-convergence or produced classes comprising less than 5% of the sample, indicating that the available data could not support additional random parameters. The limited sample size also precluded more complex modeling approaches—including three-trajectory solutions or detailed interaction analyses—and contributed to wide confidence intervals for some estimates. Nevertheless, bootstrap internal validation supported the stability of the two-trajectory solution. Third, the LCGMM analysis was necessarily restricted to patients who received IC, because only these individuals had PNI measurements at all three time points required for trajectory fitting. This restriction introduces potential selection bias, and the identified trajectories may partly reflect the nutritional impact of IC rather than universal PNI dynamics. Although an additional analysis of ΔPNI in non-IC patients yielded consistent results, this does not fully eliminate selection bias. Furthermore, while subgroup analyses confirmed consistent protective effects across all clinical strata, residual confounding by unmeasured factors cannot be entirely excluded. Fourth, we used deterministic starting values [initializing the k-class model from the estimates of the (k-1)-class model], which is the default approach in the lcmm package. Although multi-starting-value searches can provide additional reassurance against convergence to local optima, our model converged successfully and produced clinically interpretable trajectories with robust prognostic associations. Future studies with more frequent PNI measurements and larger sample sizes are warranted to refine these trajectory classifications, validate the observed class structures and explore more flexible random-effects specifications.

Several additional limitations should be acknowledged. First, its retrospective, single-center design with a modest sample size inherently limits generalizability. External validation in larger, prospective, multi-center cohorts remains essential to further confirm these findings. Second, the observed association between IC and poorer PFS should be interpreted cautiously, as it likely reflects confounding by indication, with higher-risk patients being preferentially selected for IC. Future studies should include patients both with and without IC to validate the generalizability of the trajectory findings. Third, our study population was defined by plasma EBV DNA negativity rather than tissue EBER negativity, the gold standard for confirming EBV infection. Although this definition reflects routine clinical practice and allowed a meaningful sample size, EBER data were missing for 185 patients (44.9%). Consequently, conclusions regarding EBV etiology should be interpreted with caution. Paired plasma and tissue EBV data in future studies will be important to further explore the heterogeneity among EBV DNA-negative NPC patients. Fourth, our study focused exclusively on EBV DNA-negative NPC patients; whether the identified PNI trajectories are similar or distinct in EBV DNA-positive patients remains unknown. Additionally, we did not have data on nutritional interventions (e.g., enteral nutrition support) or serial measurements of inflammatory cytokines (e.g., IL-6, CRP), which precluded mechanistic exploration of the pathways linking PNI trajectories to prognosis. Future comparative studies between EBV DNA-negative and EBV DNA-positive patients, as well as prospective studies incorporating detailed nutritional and inflammatory biomarker data, are warranted.

## 5 Conclusion

Baseline PNI is an independent prognostic factor in NPC patients with persistently undetectable plasma EBV DNA. Longitudinal PNI trajectory analysis further identifies two distinct nutritional reserve phenotypes with significantly different PFS outcomes. These findings facilitate a more precise risk stratification for this patient population and may help identify candidates for closer nutritional monitoring in future prospective studies designed to test whether early nutritional support improves clinical outcomes.

## Data Availability

The original contributions presented in the study are included in the article/Supplementary material, further inquiries can be directed to the corresponding author.
