# Supplementary material for: Dynamic prognostic nutritional index trajectories predict survival outcomes in nasopharyngeal carcinoma with persistently undetectable plasma Epstein–Barr virus DNA
Source: Front Nutr. 2026 Jul 3;13:1806364. doi: 10.3389/fnut.2026.1806364 (PMC13375451; doi:10.3389/fnut.2026.1806364)
Supplement: Supplementary file 1 [file Table_1.DOCX]

**Supplementary Methods**

**Data collection and processing**

Retrospective interrogation of the electronic laboratory records identified baseline hematological parameters, which are closest to the first diagnosis (T0). Immunoinflammatory and nutritional indices were included in this study, calculated using the following formulas: neutrophil-to-lymphocyte ratio (NLR) = neutrophil count (10^9^/L) / lymphocyte count (10^9^/L); platelet-to-lymphocyte ratio (PLR) = platelet count (10^9^/L) / lymphocyte count (10^9^/L); monocyte-to-lymphocyte ratio (MLR) = monocyte count (10^9^/L) / lymphocyte count (10^9^/L); systemic immune-inflammation index (SII) = [platelet count (10^9^/L) × neutrophil count (10^9^/L) × 10⁻^9^] / lymphocyte count (10^9^/L); systemic inflammation response index (SIRI) = [neutrophil count (10^9^/L) × monocyte count (10^9^/L) × 10⁻^9^] / lymphocyte count (10^9^/L); and pan-immune-inflammation value (PIV) = [neutrophil count (10^9^/L) × platelet count (10^9^/L) × monocyte count (10^9^/L) × 10^-18^] / lymphocyte count (10^9^/L). We further included the PNI values obtained closest to radiotherapy start (post-IC, T1), and the end of radiotherapy (post-RT, T2), as well as calculating PNI change rate, also known as delta PNI. The calculation formulas are as follows: delta PNI1 = (post-IC PNI value - baseline PNI value) / baseline PNI value × 100%; delta PNI2 = (post-RT PNI value - post-IC PNI value) / post-IC PNI value × 100%.

**Net reclassification improvement (NRI) analysis**

To evaluate the incremental prognostic value of additional factors beyond the clinical reference model (defined by T and N categories), we computed the continuous NRI at a fixed horizon of 3 years. The continuous NRI was selected for its independence from predefined risk categories and its heightened sensitivity to model improvements across the full risk continuum. Complete data were available for all model variables, obviating the need for missing data imputation. Censoring was addressed using the Kaplan-Meier estimator to ascertain event status at 3 years; patients censored before this time point were considered event-free for reclassification calculations, in accordance with standard time-to-event NRI methodology (1). Confidence intervals for the NRI were derived via bootstrap resampling with 200 replicates. A positive NRI signifies that the addition of the factors enhances risk classification relative to the reference model.

Reference

[1] Pencina MJ, D’Agostino RB Sr, Steyerberg EW. Extensions of net reclassification improvement calculations to measure usefulness of new biomarkers. *Stat Med* (2011) 30(1):11-21. doi: 10.1002/sim.4085

**Supplementary Tables**

| **Supplementary Table 1.** Proportional hazards assumption test for variables in Cox regression analysis in NPC patients with negative EBV DNA (N = 412) | | |
| --- | --- | --- |
| Characteristic | χ^2^ | P value ^a^ |
| Age, years | 0.38 | 0.535 |
| Sex | 0.31 | 0.577 |
| Histological subtype | 0.68 | 0.409 |
| T category | 1.12 | 0.291 |
| N category | 0.97 | 0.325 |
| GTV-T, Gy | 15.30 | < 0.001 |
| GTV-N, Gy | 1.96 | 0.161 |
| Induction chemotherapy | 0.47 | 0.494 |
| Concurrent chemotherapy | 1.16 | 0.281 |
| NLR | 1.90 | 0.169 |
| PLR | 0.19 | 0.665 |
| MLR | 0.17 | 0.682 |
| SII | 0.85 | 0.358 |
| SIRI | 0.95 | 0.329 |
| PIV | 0.71 | 0.398 |
| PNI | 0.00039 | 0.984 |
| ^a^ P value from Schoenfeld residual test. A P value < 0.05 indicates violation of the PH assumption.  EBV, Epstein-Barr virus; GTV-N, irradiation dose of gross tumor volume of lymph nodes; GTV-T, irradiation dose of gross tumor volume of primary nasopharyngeal tumor; MLR, monocyte-lymphocyte ratio; NLR, neutrophil-lymphocyte ratio; NPC, nasopharyngeal carcinoma; PFS, progression free survival; PIV, pan-immune-inflammation value; PLR, platelet-lymphocyte ratio; PNI, prognostic nutritional index; SII, systemic immune-inflammation index; SIRI, systemic inflammation response index. | | |

| **Supplementary Table 2**. Comparing of baseline characteristics of NPC patients with or without induction chemotherapy | | | |
| --- | --- | --- | --- |
| Characteristic | IC (n = 252) | Non-IC (n = 160) | P value |
| PNI-T1, Median (IQR) | 51.90 (48.60, 55.50) | 52.95 (49.88, 56.75) | 0.015 |
| PNI-T2, Median (IQR) | 49.90 (47.20, 53.15) | NA | NA |
| PNI-T3, Median (IQR) | 43.50 (40.40, 45.76) | 43.45 (41.00, 46.32) | 0.376 |
| Age, years, No. (%) |  |  | 0.884 |
| < 50 | 131 (52.0) | 82 (51.3) |  |
| ≥ 50 | 121 (48.0) | 78 (48.8) |  |
| Sex, No. (%) |  |  | 0.266 |
| Male | 186 (73.8) | 110 (68.8) |  |
| Female | 66 (26.2) | 50 (31.3) |  |
| Histological subtype, No. (%) |  |  | 0.341 |
| Nonkeratinizing carcinoma | 246 (97.6) | 159 (99.4) |  |
| Keratinizing carcinoma | 6 (2.4) | 1 (0.6) |  |
| EBER status |  |  | 0.020 |
| Positive | 129 (51.2) | 74 (46.3) |  |
| Negative | 20 (7.9) | 4 (2.5) |  |
| Missing | 103 (40.9) | 82 (51.3) |  |
| T category, No. (%) |  |  | < 0.001 |
| T1-2 | 67 (26.6) | 150 (93.8) |  |
| T3-4 | 185 (73.4) | 10 (6.3) |  |
| N category, No. (%) |  |  | < 0.001 |
| N0-1 | 142 (56.4) | 156 (97.5) |  |
| N2-3 | 110 (43.7) | 4 (2.5) |  |
| GTV-T, Gy, Median (IQR) | 69.96 (69.96, 70.00) | 69.75 (69.75, 69.96) | < 0.001 |
| GTV-N, Gy, Median (IQR) | 69.96 (69.30, 70.00) | 69.75 (66.65, 69.75) | < 0.001 |
| Number of cycles of IC, cycles, No. (%) |  |  | < 0.001 |
| 0 | 0 (0.0) | 160 (100.0) |  |
| 1-3 | 177 (70.2) | 0 (0.0) |  |
| > 3 | 75 (29.8) | 0 (0.0) |  |
| Concurrent chemotherapy, No. (%) |  |  | 0.275 |
| No | 92 (36.5) | 67 (41.9) |  |
| Yes | 160 (63.5) | 93 (58.1) |  |
| NLR, Median (IQR) | 1.96 (1.48, 2.84) | 1.89 (1.50, 2.42) | 0.313 |
| PLR, Median (IQR) | 122.03 (94.40, 163.98) | 122.02 (99.43, 152.19) | 0.886 |
| MLR, Median (IQR) | 0.20 (0.15, 0.27) | 0.19 (0.15, 0.25) | 0.183 |
| SII, Median (IQR) | 462.06 (315.28, 658.55) | 461.07 (350.92, 622.97) | 0.578 |
| SIRI, Median (IQR) | 0.80 (0.52, 1.20) | 0.68 (0.51, 1.00) | 0.114 |
| PIV, Median (IQR) | 185.30 (98.09, 283.33) | 166.34 (110.32, 255.57) | 0.539 |
| Continuous variables were represented as median with IQR; while categorical data were represented as absolute values and proportions. EBER, Epstein-Barr virus-encoded small RNA; EBV DNA, Epstein-Barr virus DNA; GTV-N, irradiation dose of gross tumor volume of lymph nodes; GTV-T, irradiation dose of gross tumor volume of primary nasopharyngeal tumor; IC, induction chemotherapy; IQR, interquartile range; MLR, monocyte-lymphocyte ratio; NLR, neutrophil-lymphocyte ratio; NPC, nasopharyngeal carcinoma; PFS, progression free survival; PIV, pan-immune-inflammation value; PLR, platelet-lymphocyte ratio; PNI, prognostic nutritional index; SII, systemic immune-inflammation index; SIRI, systemic inflammation response index. | | | |

| **Supplementary Table 3.** Proportional hazards assumption test for variables in Cox regression analysis in NPC patients with negative EBV DNA receiving induction chemotherapy (n = 252) | | |
| --- | --- | --- |
| Characteristic | χ^2^ | P value ^a^ |
| Age, years | 0.12 | 0.727 |
| Sex | 0.91 | 0.341 |
| Histological subtype | 0.39 | 0.535 |
| T category | 2.41 | 0.121 |
| N category | 0.41 | 0.520 |
| GTV-T, Gy | 19.50 | < 0.001 |
| GTV-N, Gy | 1.66 | 0.197 |
| Number of cycles of induction chemotherapy, cycles | 3.89 | 0.049 |
| Concurrent chemotherapy | 2.37 | 0.124 |
| NLR | 1.14 | 0.285 |
| PLR | 0.0011 | 0.974 |
| MLR | 2.61 | 0.106 |
| SII | 0.36 | 0.547 |
| SIRI | 2.72 | 0.099 |
| PIV | 0.71 | 0.400 |
| PNI | 0.04 | 0.849 |
| ^a^ P value from Schoenfeld residual test. A P value < 0.05 indicates violation of the PH assumption.  EBV, Epstein-Barr virus; GTV-N, irradiation dose of gross tumor volume of lymph nodes; GTV-T, irradiation dose of gross tumor volume of primary nasopharyngeal tumor; MLR, monocyte-lymphocyte ratio; NLR, neutrophil-lymphocyte ratio; NPC, nasopharyngeal carcinoma; PFS, progression free survival; PIV, pan-immune-inflammation value; PLR, platelet-lymphocyte ratio; PNI, prognostic nutritional index; SII, systemic immune-inflammation index; SIRI, systemic inflammation response index. | | |

| **Supplementary Table 4.** Identification of prognostic factors of PFS in EBV DNA-negative NPC patients receiving induction chemotherapy by univariate and multivariate Cox regression analysis | | | | | | |
| --- | --- | --- | --- | --- | --- | --- |
| Characteristic | Univariate | |  | Multivariate | |  |
|  | HR (95% CI) | P value |  | HR (95% CI) | P value |  |
| Age, years |  |  |  |  |  |  |
| < 50 | 1.00 (Reference) |  |  | 1.00 (Reference) |  |  |
| ≥ 50 | 2.14 (1.13–4.07) | 0.020 |  | 1.84 (0.94–3.61) | 0.074 |  |
| Sex |  |  |  |  |  |  |
| Male | 1.00 (Reference) |  |  |  |  |  |
| Female | 1.21 (0.63–2.33) | 0.565 |  |  |  |  |
| Histological subtype |  |  |  |  |  |  |
| Nonkeratinizing carcinoma | 1.00 (Reference) |  |  | 1.00 (Reference) |  |  |
| Keratinizing carcinoma | 9.26 (3.22–26.60) | < 0.001 |  | 7.35 (2.21–24.46) | 0.001 |  |
| T category |  |  |  |  |  |  |
| T1-2 | 1.00 (Reference) |  |  |  |  |  |
| T3-4 | 0.77 (0.40–1.49) | 0.441 |  |  |  |  |
| N category |  |  |  |  |  |  |
| N0-1 | 1.00 (Reference) |  |  | 1.00 (Reference) |  |  |
| N2-3 | 1.93 (1.04–3.58) | 0.037 |  | 2.17 (1.15–4.09) | 0.016 |  |
| GTV-T, Gy | 0.14 (0.02–1.33) | 0.087 |  |  |  |  |
| GTV-N, Gy | 1.00 (1.00–1.00) | 0.466 |  |  |  |  |
| Number of cycles of induction chemotherapy, cycles |  |  |  |  |  |  |
| ≤ 3 | 1.00 (Reference) |  |  |  |  |  |
| > 3 | 0.92 (0.46–1.83) | 0.809 |  |  |  |  |
| Concurrent chemotherapy |  |  |  |  |  |  |
| No | 1.00 (Reference) |  |  |  |  |  |
| Yes | 1.80 (0.86–3.76) | 0.121 |  |  |  |  |
| NLR | 1.24 (0.99–1.55) | 0.058 |  |  |  |  |
| PLR | 1.01 (1.01–1.01) | 0.014 |  | 1.00 (1.00–1.01) | 0.190 |  |
| MLR | 10.05 (0.66–152.60) | 0.096 |  |  |  |  |
| SII | 1.01 (1.01–1.01) | 0.018 |  | 1.00 (1.00–1.00) | 0.873 |  |
| SIRI | 1.11 (0.77–1.61) | 0.572 |  |  |  |  |
| PIV | 1.00 (1.00–1.00) | 0.191 |  |  |  |  |
| PNI | 0.91 (0.85–0.96) | 0.001 |  | 0.93 (0.88–0.99) | 0.038 |  |
| EBV, Epstein-Barr virus; GTV-N, irradiation dose of gross tumor volume of lymph nodes; GTV-T, irradiation dose of gross tumor volume of primary nasopharyngeal tumor; MLR, monocyte-lymphocyte ratio; NLR, neutrophil-lymphocyte ratio; NPC, nasopharyngeal carcinoma; PFS, progression free survival; PIV, pan-immune-inflammation value; PLR, platelet-lymphocyte ratio; PNI, prognostic nutritional index; SII, systemic immune-inflammation index; SIRI, systemic inflammation response index. | | | | | | |

| **Supplementary Table 5.** Collinearity diagnostics for variables included in the final multivariable Cox regression model in NPC patients with negative EBV DNA (N = 412) | |
| --- | --- |
| Characteristic | VIF ^a^ |
| Age, years | 1.04 |
| Histological subtype | 1.01 |
| N category | 1.27 |
| Induction chemotherapy | 1.27 |
| PNI | 1.06 |
| ^a^ A VIF > 5 typically indicates significant collinearity.  EBV, Epstein-Barr virus; PNI, prognostic nutritional index; VIF, variance inflation factor. | |

| **Supplementary Table 6.** Collinearity diagnostics for variables included in the final multivariable Cox regression model in NPC patients with negative EBV DNA receiving induction chemotherapy (n = 252) | |
| --- | --- |
| Characteristic | VIF ^a^ |
| Age, years | 1.07 |
| Histological subtype | 1.11 |
| N category | 1.03 |
| PLR | 1.52 |
| SII | 1.56 |
| PNI | 1.18 |
| ^a^ A VIF > 5 typically indicates significant collinearity.  EBV, Epstein-Barr virus; PLR, platelet-lymphocyte ratio; PNI, prognostic nutritional index; VIF, variance inflation factor. | |

| **Supplementary Table 7**. Baseline characteristics of NPC patients in different PNI trajectory subgroups | | | |
| --- | --- | --- | --- |
| Characteristic | Low Nutritional Reserve Type (n = 184) | High Nutritional Reserve Type (n = 68) | P value |
| PNI-T1, Median (IQR) | 50.30 (47.20, 52.60) | 58.00 (56.32, 60.20) | < 0.001 |
| PNI-T2, Median (IQR) | 48.65 (46.35, 51.05) | 54.15 (51.70, 56.28) | < 0.001 |
| PNI-T3, Median (IQR) | 42.95 (39.98, 45.40) | 44.85 (42.55, 46.75) | < 0.001 |
| Age, years, No. (%) |  |  | 0.030 |
| < 50 | 88 (47.8) | 43 (63.2) |  |
| ≥ 50 | 96 (52.2) | 25 (36.8) |  |
| Sex, No. (%) |  |  | 0.012 |
| Male | 128 (69.6) | 58 (85.3) |  |
| Female | 56 (30.4) | 10 (14.7) |  |
| Histological subtype, No. (%) |  |  | 0.912 |
| Nonkeratinizing carcinoma | 179 (97.3) | 67 (98.5) |  |
| Keratinizing carcinoma | 5 (2.7) | 1 (1.5) |  |
| EBER status |  |  | 0.476 |
| Positive | 98 (53.3) | 31 (45.6) |  |
| Negative | 15 (8.2) | 5 (7.4) |  |
| Missing | 71 (38.6) | 32 (47.1) |  |
| T category, No. (%) |  |  | 0.729 |
| T1-2 | 50 (27.2) | 17 (25.0) |  |
| T3-4 | 134 (72.8) | 51 (75.0) |  |
| N category, No. (%) |  |  | 0.180 |
| N0-1 | 99 (53.8) | 43 (63.2) |  |
| N2-3 | 85 (46.2) | 25 (36.8) |  |
| GTV-T, Gy, Median (IQR) | 69.96 (69.96, 70.00) | 69.96 (69.96, 70.00) | 0.888 |
| GTV-N, Gy, Median (IQR) | 69.96 (69.30, 70.00) | 69.96 (68.78, 70.00) | 0.803 |
| Number of cycles of induction chemotherapy, cycles, No. (%) |  |  | 0.584 |
| ≤ 3 | 131 (71.2) | 46 (67.7) |  |
| > 3 | 53 (28.8) | 22 (32.4) |  |
| Concurrent chemotherapy, No. (%) |  |  | 0.259 |
| No | 71 (38.6) | 21 (30.9) |  |
| Yes | 113 (61.4) | 47 (69.1) |  |
| Continuous variables were represented as median with IQR; while categorical data were represented as absolute values and proportions. EBER, Epstein-Barr virus-encoded small RNA; EBV DNA, Epstein-Barr virus DNA; GTV-N, irradiation dose of gross tumor volume of lymph nodes; GTV-T, irradiation dose of gross tumor volume of primary nasopharyngeal tumor; IQR, interquartile range; MLR, monocyte-lymphocyte ratio; NLR, neutrophil-lymphocyte ratio; NPC, nasopharyngeal carcinoma; PFS, progression free survival; PIV, pan-immune-inflammation value; PLR, platelet-lymphocyte ratio; PNI, prognostic nutritional index; SII, systemic immune-inflammation index; SIRI, systemic inflammation response index. | | | |

| **Supplementary Table 8**. Bootstrap internal validation of LCGMM trajectory classification | |
| --- | --- |
| Metric | Value |
| Bootstrap iterations | 200 |
| Model convergence rate | 200/200 (100%) |
| Replicates supporting 2‑trajectory solution | 100% |
| Proportion of high reserve group (bootstrap mean, 95%CI) | 39.9% (17.8%–61.9%) |
| Bootstrap‑adjusted HR, 95% Bootstrap CI (high vs. low reserve) | 0.37 (0.15–0.91) |
| LCGMM, latent class growth mixed model. | |

| **Supplementary Table 9.** Incremental prognostic value of PNI trajectory for PFS in NPC patients with negative EBV DNA receiving induction chemotherapy | | | | | | |
| --- | --- | --- | --- | --- | --- | --- |
| Model | Variables | C-index (95% CI) | NRI (95% CI) | AIC | Likelihood ratio test P value | |
| Model 1 | T + N category | 0.56 (0.52–0.61) | Reference | 431.10 | 0.104 | |
| Model 3 | T + N category + PNI trajectory | 0.63 (0.59–0.68) | 0.16 (0.01–0.30) | 427.92 | 0.021 | |
| Model 4 | T + N category + SIRI + PIV | 0.59 (0.54–0.64) | 0.03 (0.00–0.10) | 433.95 | 0.224 | |
| Model 5 | T + N category + SIRI + PIV + PNI trajectory | 0.66 (0.61–0.70) | 0.03 (0.00–0.10) | 431.03 | 0.060 | |
| AIC, **Akaike Information Criterion;** C-index, Harrell’s concordance index; EBV DNA, Epstein-Barr virus DNA; NPC, nasopharyngeal carcinoma; NRI, **net reclassification improvement**; PFS, progression free survival; PNI, prognostic nutritional index. | | | | | | |
|  |  |  |  |  |  |  |

**Supplementary Figures**

**
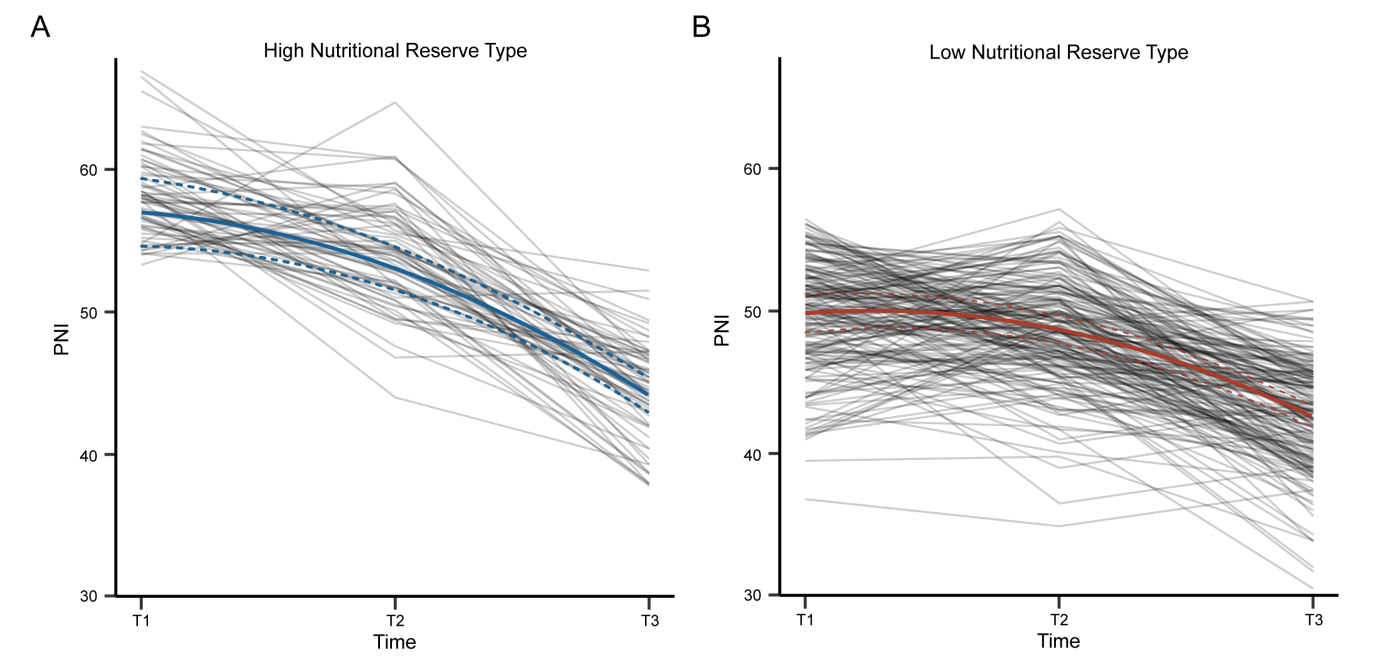
**

**Supplementary Figure 1.** Individual PNI trajectories for patients (gray lines) with overlaid fitted class trajectories (colored bold lines: blue for high nutritional reserve, red for low nutritional reserve).

**
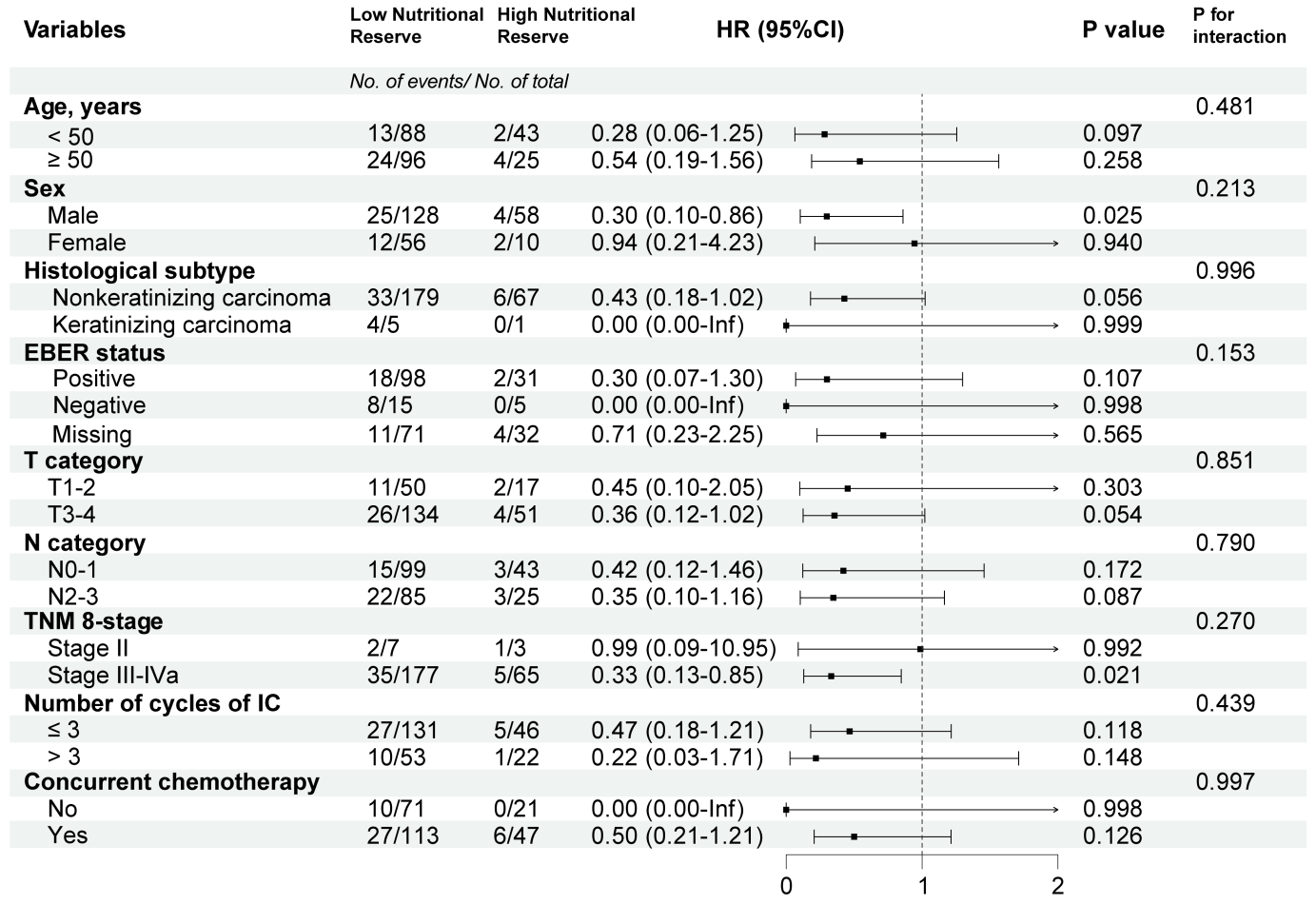
**

**Supplementary Figure 2.** Subgroup analyses of the association between PNI trajectory and PFS. EBER, Epstein-Barr virus-encoded small RNA; IC,induction chemotherapy; PFS, progression free survival; PNI, prognostic nutritional index.
